# Regulation of Hepatitis B Virus Replication by Modulating Endoplasmic Reticulum Stress (ER-Stress)

**DOI:** 10.1155/2024/9117453

**Published:** 2024-08-30

**Authors:** Md. Golzar Hossain, Keiji Ueda

**Affiliations:** ^1^ Department of Microbiology and Hygiene Bangladesh Agricultural University, Mymensingh 2202, Bangladesh; ^2^ Division of Virology Department of Microbiology and Immunology Graduate School of Medicine Osaka University, Osaka, Japan

## Abstract

Hepatitis B virus (HBV), resistant to several antiviral drugs due to viral genomic mutations, has been reported, which aggravates chronic infection and leads to hepatocellular carcinoma. Therefore, host cellular factors/signaling modulation might be an alternative way of treatment for drug-resistant HBV. Here, we investigated the viral protein expression, replication, and virion production using endoplasmic reticulum (ER) stress-modulating chemicals, tunicamycin (an ER-stress inducer), and salubrinal (an ER-stress inhibitor). We found that ER-stress could be induced by HBV replication in transfected HepG2 cells as well as by tunicamycin as demonstrated by dual luciferase assay. HBV intracellular core-associated DNA quantified by qPCR has been significantly increased by tunicamycin in transfected HepG2 cells. Inversely, intracellular core associated and extracellular particle DNA has been significantly decreased in a dose-dependent manner in salubrinal-treated HepG2 cells transfected with HBV-replicating plasmid pHBI. Similar results were found in stably HBV-expressing hepatoblastoma (HB611) cells treated with salubrinal. However, increased or decreased ER-stress by tunicamycin or salubrinal treatment, respectively, has been confirmed by expression analysis of grp78 using Western blot. In addition, Western blot results demonstrated that the expression of HBV core protein and large HBsAg is increased and decreased by tunicamycin and salubrinal, respectively. In conclusion, the sal-mediated inhibition of the HBV replication and virion production might be due to the simultaneous reduction of core and large HBsAg expression and maintaining the ER homeostasis. These results of HBV replication regulation by modulation of ER-stress dynamics would be useful for designing/identifying anti-HBV drugs targeting cellular signaling pathways.

## 1. Introduction

Hepatitis B (HB) is a noxious infectious disease of the liver caused by the hepatitis B virus (HBV). It is one of the significant public health problems worldwide [1]. HB might be both acute and chronic and infected patients are at high risk due to hepatocellular carcinoma (HCC) [2]. HBV is an enveloped virus and consists of an icosahedral nucleocapsid. The 3.2 kb partially double-stranded DNA of HBV encodes four overlapping open reading frames (ORFs), namely, polymerase (P), surface or envelope (S), core (C), and X protein (X) [3, 4]. The P protein has reverse transcriptase activity which plays a vital role in viral replication. Therefore, most of the anti-HBV drugs have been used to target P to reduce and/or stop viral replication [5]. The C protein is self-assembled to encapsidate the pgRNA to form a viral nucleocapsid [6]. The surface proteins; large, middle, and small HBsAg assembled with the nucleocapsid in the ER-Golgi/MVB to fulfill the viral replication and secreted into the extracellular space [3, 7]. The X is a nonstructural multifunctional protein supposed to be responsible for the development of HCC by regulating many cellular signaling pathways [8, 9]. However, almost all the viral proteins interacted with many cellular factors to complete its replication cycle and thereby affect host disease progression [3, 10].

The vaccine has been used to prevent HBV infection. But vaccination failure may occur due to viral genomic mutations including many unknown factors [11–16]. Anti-HBV drugs are used to reduce and/or stop viral replication in infected patients. Severe side effects may occur due to long-term treatment and could not be cured completely [17–20]. Moreover, mutations may occur in the P protein, which leads to the development of drug-resistant HBV [21–23]. Interferon-*α* (IFN-*α*) is the immunomodulatory drug for HBV treatment. Though IFN is effective against nucleot(s)ide analogues (NAs)-resistant HBV, it could not able to clear the infection completely and has adverse side effects [18, 24].

Novel anti-HBV drug screening has been continuing though having many limitations [25–27]. Therefore, researchers have been trying to regulate the host cellular signaling to reduce and/or stop viral replication [3, 28–31]. Endoplasmic reticulum (ER)-stress is one of the important signaling pathways involved in liver diseases and may be a therapeutic target for treatment [32].

ER is a large organelle that plays important roles in protein synthesis, folding, modification, transport of protein, and calcium storage [33]. ER membranes are connected with the outer membrane of the nucleus. On the other hand, the outer mitochondrial membrane can associate with ER membrane to form a mitochondria-associated ER membrane (MAM) through which calcium signaling communication occurs between OMM and ER [3, 34]. However, the properly folded proteins are transported from ER to Golgi bodies, and unfolded protein response (UPR) occurs as ER-stress due to unfolded or misfolded proteins [33]. UPR or ER-stress might be induced by many viruses including HBV [35–39]. HBV-induced ER-stress is supposed to be responsible for liver disease progression [32, 35, 40]. HBV surface proteins are transmembrane proteins and activate UPR [41, 42]. Li et al. reported that X protein is localized into the lumen of ER and modulates ER-stress [37, 43]. ER stress is also upregulated by the naturally mutated HBV core protein involved in disease progression [44]. Several hypotheses indicate that HBV nucleocapsid assembled with surface proteins in ER-Golgi/MVB [7, 45]. However, information regarding the HBV replication strategy by modulating ER stress using chemical agents is limited. Tunicamycin (tm) can induce ER-stress by unfolded protein response (UPR) [46, 47]. On the other hand, salubrinal (sal) protects the cells from ER-stress [48, 49]. Sal selectively inhibits the dephosphorylation and maintains the phosphorylation state of eukaryotic translation initiation factor 2*α* (eIF2*α*), which leads to the suppression of protein translation and synthesis to maintain ER homeostasis [49]. Therefore, in this study, we analyzed the effect modulation of ER-stress by using tm and sal on HBV replication. We found that tm aggravated the HBV-induced ER-stress and upregulated core, large HBsAg, and HBV replication whereas sal downregulated core, large HBsAg, and HBV replication in both transiently transfected HepG2 and stably replicating HB611 cells.

## 2. Materials and Methods

### 2.1. Plasmids and Cell Lines

HBV-replicating plasmid pHBI containing 1.3-fold overlapped viral genome (X01587.1) in the pBR322 vector [50]. ER-stress detector plasmids (pERAI-Luc) and pRL-TK (Control) were a kind gift from Professor Toru Okamoto [46]. HepG2 cells originally obtained from ATCC (ATCC® HB-8065™) were maintained in high glucose Dulbecco's modified Eagle's medium (DMEM) (Nacalai Tesque) supplemented with 10% fetal bovine serum (Equitech-Bio Inc.) [51]. HB611 cells [52] were maintained similarly using low glucose DMEM containing 0.5 mg/mL of neomycin (G 418 Disulfate Aqueous Solution, product number-09380-44, Nakalai Tesque, Inc.). All the media contained 100 U/mL penicillin G, 100 *μ*g/mL streptomycin, and 0.25 *μ*g/mL amphotericin B, and cells are cultured at 37°C in an atmosphere of 5% CO_2_ [50].

### 2.2. Transfection

HepG2 cells (1-2 × 10^5^ cells/well) were seeded on a 24-well collagen-coated plate and incubated for 24 hours for luciferase assay. The cells were then cotransfected with pERAI-Luc, pRL-TK, and pHBI using GenJet™ In Vitro DNA Transfection Reagent (SignaGen® Laboratories) following manufacturer's protocol. The medium was replaced with fresh medium ∼6 hours after transfection. The cells were further incubated for 24 hours for luciferase assay. To observe the effect of sal or tm on HBV replication, HepG2 cells (1-2 × 10^5^ cells/well) were seeded on a 24-well collagen-coated plate and transfected similarly with pHBI plasmid.

### 2.3. Drug Treatment

HB611 cells were seeded (0.5–1.0 × 10^5^ cells/well) on a 24-well plate and incubated for 24 hours. Then, the medium was replaced with sal (sc-202332, Santa Cruz Biotechnology) containing medium and incubated for 3 days. The medium was further replaced at day 3 of treatment with fresh sal-containing medium and incubated further 3 days (Total 6 days of posttreatment) for analysis. The HepG2 cells, ∼6 hours after transfection, were treated similarly with either sal or tm and incubated for a total of 6 days. Entecavir (ETV), a known HBV replication inhibitor, was used as a control in the study and standardized to 20 nM based on our previous study [53]. Then, the virus-containing culture supernatants were collected and passed through a 0.43 *μ*m filter (Millex®) and subjected to DNA extraction and ELISA. The cells were washed with phosphate buffer solution (PBS) and transferred for DNA extraction or Western blot analysis.

### 2.4. Cell Viability Assay

HepG2 cells were seeded on 96-well collagen-coated (3 × 10^4^ cells/well) clear bottom white plate (Corning) with 100 *μ*l culture medium and incubated for 24 hours. The cells were then treated with the mentioned concentration of tm or sal as previously described. A 100 *μ*l of CellTiterGlo® luminescent cell viability assay solution (Promega) was poured into the culture medium and incubated at 37°C for 15 min. The luminescence was measured using GloMax® GM3000 (Promega) according to the manufacturer's guidelines.

### 2.5. Luciferase Assay

Luciferase assay was conducted using Dual-Luciferase® Reporter Assay System (Promega, #E1960). Working reagents were prepared according to the manufacturer's recommended guidelines. Transfected cells were washed with PBS in a cultured plate and 100 *μ*l of passive lysis buffer (PLB) was added. The plate containing monolayer cells and PLB was incubated for 15 minutes at room temperature with gentle shaking. The lysed cells were collected into the 1.5 ml Eppendorf tube and debris was removed by centrifugation at 10000 × g for 1 minute. Then, firefly and renilla luciferase were measured using a luminometer according to manufacturer's protocol.

### 2.6. Enzyme-Linked Immunosorbent Assay (ELISA) and Western Blot

HBsAg and HBeAg were detected using commercially available ELISA kits (HBsAg ELISA rapid II, Beacle Inc., Kyoto, Japan, and HBeAg ELISA Kit, Bioneovan, Beijing, China). The supernatants either from transfected HepG2 cells or HB611 cells were diluted in PBS, and ELISA was performed according to the previously used protocol [3, 54]. The cells were lysed with 50 mM NaH_2_PO_4_ (pH: 8.0), 300 mM NaCl, 0.1% NP40, and a complete mammalian protease inhibitor (Sigma P8849; 1 : 1000 dilution). The prepared lysates were mixed with 5X SDS-PAGE sample buffer and boiled for 5 min at 100°C. The protein separated by SDS-polyacrylamide gel (Bio-Rad) electrophoresis was then transferred to a PVDF membrane (Cat #162–0177; Bio-Rad) and blocked with 5% skim milk in TBS-T [54]. Mouse monoclonal anti-HBV pre-S1 antibody (mouse mono-1, Catalog No. BCL-AB-01; Beacle, Inc.), rabbit polyclonal anti-HBc Ab (Anti-HBcAg Antibody, Poly, Catalog No. BCL-ABPC-01; Beacle, Inc.), rabbit polyclonal anti grp78 Ab (Anti-HSPA5, product number-HPA038845, Sigma), and mouse monoclonal anti-*β*-tubulin Ab (Sigma Cat# T5201) were used as a primary antibody to detect specific proteins as described previously [3, 54]. Horseradish peroxidase (HRP)-labeled goat polyclonal anti-rabbit and anti-mouse immunoglobulins (Dako, Denmark) were used as secondary antibodies. The specific proteins were visualized as described previously [54, 55].

### 2.7. DNA Extraction and Quantitative Real-Time PCR (qPCR)

The extra-virion DNA in the supernatant was digested with DNase I (Takara; 1 : 500 dilution) treatment and the reaction was stopped using EDTA as previously described [55]. The DNA from the concentrated virus particles was extracted as described previously using a glycogen as a carrier [55]. For intracellular core DNA extraction, the cell membrane was ruptured using a hypotonic buffer (50 mM Tris-HCl pH 7.4, 1 mM EDTA, and 1% Nonidet P-40). Then, the cytoplasmic fraction containing HBV particles was collected by centrifuging for 15 minutes at 17,000 × g. The samples were treated with 2 *μ*L (10 unit) DNase I and 5 *μ*L (0.5 *μ*g/ml) RNase A (Takara-Clontech) in the presence of 5 mM MgCl_2_ and CaCl_2_ for at least 3 hours at 37°C. The reaction is then stopped by adding 10 mM of EDTA and EGTA. The HBV core particles are lysed by 50 mM NaCl, 1% SDS, and 0.2 mg/mL proteinase K (Roche, Switzerland) and incubated at 56°C for at least 3 hours. The samples were spun down briefly and glycogen was added as a carrier. Then, the HBV DNA was isolated by phenol-chloroform isopropanol extraction followed by ethanol precipitation and rinsing with 70% ethanol, dried, and finally dissolved in Tris-EDTA buffer (TE; 10 mM Tris (pH:8.0) and 1 mM EDTA). The HBV DNA was quantified by qPCR with a specific primer set (each at 200 nM) of HBs F2: 5′-CTTCATCCTGCTGCTATGCCT-3′ and HBs R2: 5′- AAAGCCCAGGATGATGGGAT-3. The quantification was carried out using SYBR® Green Master Mix (Thermo Fisher Scientific, United States) in QuantStudioTM 6 Flex (Thermo Fisher Scientific, United States) following the manufacturer's guidelines. Triplicate reactions for each sample were performed and the average values were calculated according to the standard line [55].

### 2.8. Statistical Analysis

Statistical significance of different data was determined using a paired *t*-test; a *p* value of less than 0.05 (*p* < 0.05) was considered statistically significant, and a *p* value of less than 0.001 (*p* < 0.001) was considered highly significant. Each experiment was performed at least three times and the results were presented as the mean ± standard deviation of the mean (SD).

## 3. Results

### 3.1. HBV Induced ER-Stress in HepG2 Cells

**Figure 1:**
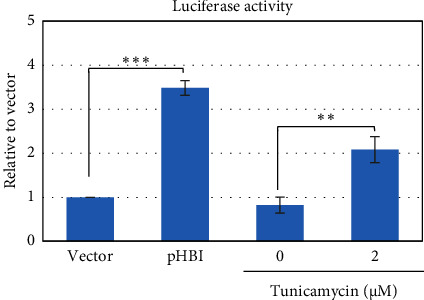
Effect of HBV replication on ER stress. HepG2 cells were cotransfected with pERAI-Luc and pRL-TK with vector or pHBI. Only pERAI-Luc and pRL-TK cotransfected cells were treated with the indicated concentration of tm. All the cells were incubated for further 24 hours and luciferase activity was measured. The data are presented with error bars representing SD; ^∗∗^ represents statistically significant (*p* < 0.01) and ^∗∗∗^ indicates (*p* < 0.001).

ER-stress might be induced by exogenous envelope proteins and by mutated core and X protein of HBV [37, 42–44]. Therefore, we first analyzed the induction of ER-stress by HBV replication in HepG2 cells cotransfected with pERAI-Luc and pRL-TK and with an HBV replication-competent plasmid, pHBI. Results demonstrated that luciferase activity is significantly upregulated in cells transfected with pHBI compared with empty vector (Figure 1). Furthermore, tunicamycin itself is also able to induce ER-stress by unfolded protein response (UPR) (Figure 1) [46, 47]. These results further confirmed that ER-stress could be induced by the HBV replication in transfected HepG2 cells.

### 3.2. Tunicamycin-Induced Elevated ER-Stress Upregulates HBV Replication

**Figure 2:**
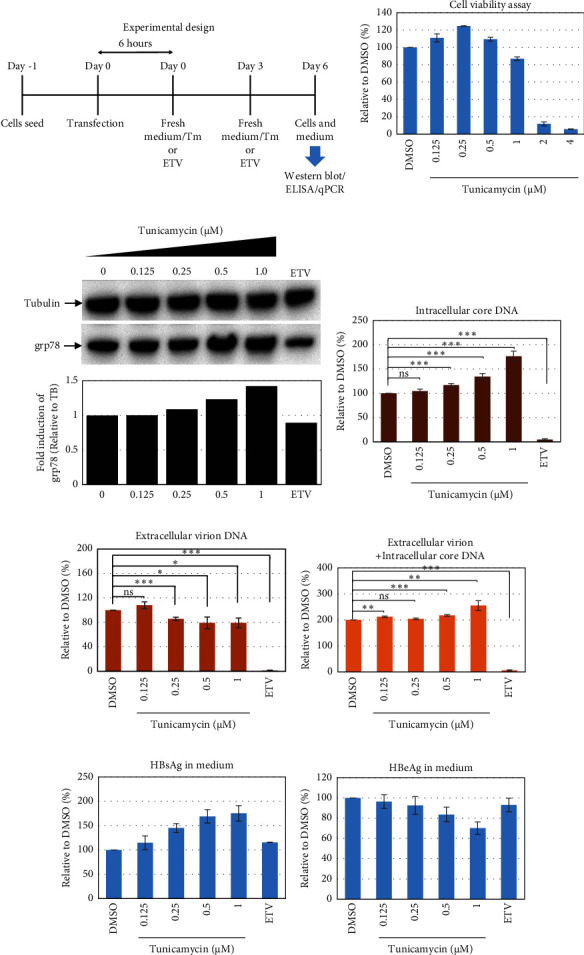
Effect of tunicamycin (tm) on the HBV replication and virion production in HepG2 cells. HepG2 cells were seeded, transfected with the pHBI, and treated with tm at indicated concentration. Cells and medium were collected after 6 days of post-transfection/tm treatment for further analysis. (a) Cell viability assay. (b) Experimental design. (c) Western blot analysis from the extracted proteins from cells using indicated antibodies. Graph shows the relative expression of grp78 relative to tubulin (TB) generated from the Western blot band intensities using ImageJ (https://ij.imjoy.io/). (d) Intracellular core associated DNA quantification by qPCR. (e) The extracellular particle-associated DNA quantification by qPCR. (f) Total of intracellular core and extracellular particle-associated DNA. (g) HBsAg and (h) HBeAg ELISA from the medium of the cultured cells. Each of the experiments was conducted at least three independent times and results were presented as the mean ± SD. ^∗^ represents statistically significant (*p* < 0.05), ^∗∗^ indicates (*p* < 0.01), and ^∗∗∗^ indicates (*p* < 0.001).

**Figure 3:**
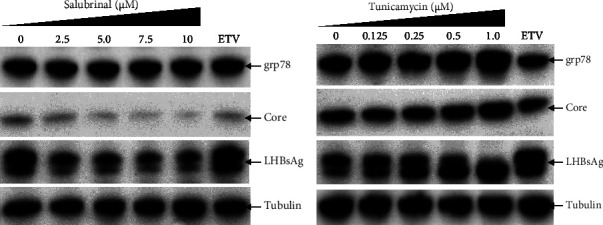
Effect of sal and tm on the HBV core and large HBsAg expression in HepG2 cells. HepG2 cells were seeded, transfected with the pHBI, and treated with sal or tm at the indicated concentration. Cells and medium were collected after 6 days of post-transfection and sal or tm treatment. Total proteins were extracted, separated by SDS-PAGE, and Western blot was performed using indicated antibodies. (a) Western blot analysis from sal-treated cells. (b) Western blot analysis from tm-treated cells. LHBsAg is a glycosylated protein and, therefore, both bands correspond to LHBsAg. The experiment was performed at least three independent times and one representative figure is presented.

To test whether ER-stress is beneficial or harmful for HBV replication, we transfected the HepG2 cells with pHBI and treated them with tunicamycin (tm) at different concentrations (Figure 2(b)). The tm did not show any significant cytotoxicity to the cells up to 1.0 *μ*M, and cell viability has been increased a little with 0.125–0.5 *μ*M of concentration (Figure 2(a)). The tm induced the cellular grp78/BIP expression as shown by Western blot, which is the marker of ER-stress (Figure 2(c)) [47]. On the other hand, the treatment with tm significantly increased the HBV intracellular core-associated DNA in a dose-dependent manner which is correlated with the elevated level of ER-stress (Figures 2(c) and 2(d)). Surprisingly, the extracellular viron DNA was decreased in the cells treated with 0.25–1 *μ*M of tm, though a little increase has been observed at 0.125 *μ*M (Figure 2(e)). The sum of intracellular core associated and extracellular virion DNA has been increased from the cells treated with tm (Figure 2(f)). The tm induced the ER-stress by UPR and removed the N-linked glycosylation of the proteins [56–58]. Besides, the removal of N-linked glycosylation of HBsAg drastically reduced the HBV virion reduced [59, 60]. Accordingly, tm removed the glycosylation isoform of HBsAg in a dose-dependent manner, which may reduce the release of HBV though intracellular core production has been increased (Figures 2(d) and 3(b)). These results suggested that HBV replication and virion production might be upregulated by the elevating ER-stress. However, the secretory HBeAg and HBsAg in the medium of the tm-treated cells seem to be reduced and increased, respectively (Figure 2(g) and 2(h)). The expression profile of HBeAg, HBsAg, and replication and virion production from the transfected cells treated with ETV strongly correlated with the previous findings (Figure 2) [53].

### 3.3. Salubrinal Inhibited HBV Replications in Transfected HepG2 Cells

**Figure 4:**
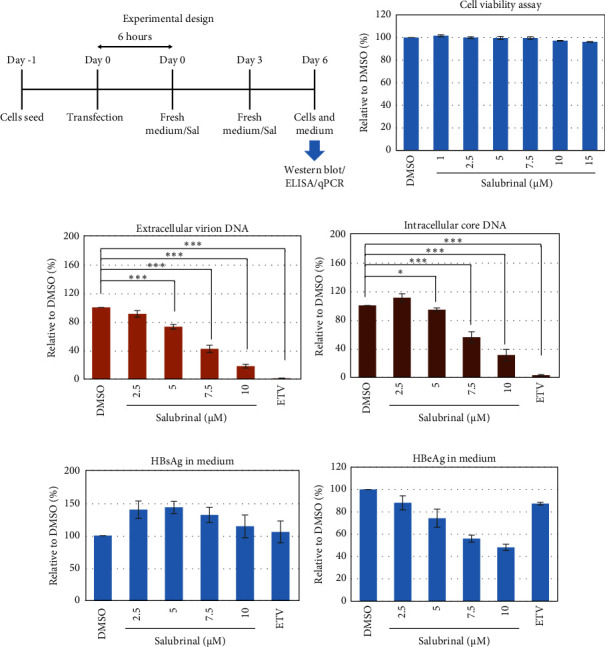
Effect of salubrinal (sal) on the HBV replication and virion production in HepG2 cells. HepG2 cells were seeded, transfected with the pHBI, and treated with sal at the indicated concentration. Cells and medium were collected after 6 days of post-transfection/sal treatment for further analysis. (a) Cell viability assay. (b) Experimental design. (c) The extracellular particle-associated DNA quantification by qPCR. (d) Intracellular core associated DNA quantification by qPCR. (e) HBsAg and (f) HBeAg ELISA from the medium of the cultured cells. Each of the experiments was conducted at least three independent times and results were presented as mean ± SD. ^∗^ represents statistically significant (*p* < 0.05) and ^∗∗∗^ indicates (*p* < 0.001).

Salubrinal (sal) is a well-known ER-stress inhibitor that maintains ER homeostasis by selectively inhibiting the dephosphorylation of eIF2*α* [49]. Therefore, the HBV replication has been investigated in the HepG2 transfected cells treated with sal. The intracellular core-associated DNA was extracted and quantified from the sal-treated pHBI transfected HepG2 cells using qPCR (Figure 4(b)). Treatment of sal significantly reduced the intracellular core-associated DNA of HBV in a dose-dependent manner (Figure 4(c)). Once the intracellular core particle is reduced, the virion assembly and release should be affected. As expected, extracellular virion production from sal-treated cells was also significantly reduced in a dose-dependent manner compared with the nontreated cells (Figure 4(d)). However, ELISA results showed that the expression of HBsAg increased by treatment of sal (Figure 4(e)). Moreover, HBeAg significantly downregulated in the transfected HepG2 cells treated with sal dose dependently (Figure 4(f)). As previously reported, our experiment also showed that intracellular core-associated and extracellular viral DNA was significantly reduced by ETV without affecting the expression of HBeAg and HBsAg (Figure 4(f)) [53, 61]. However, HepG2 cells were viable more than 85% up to the concentration of 10 *μ*M of sal (Figure 4(a)). Taken together, sal significantly inhibited HBeAg secretion, HBV replication, and virion production in transfected cells.

### 3.4. HBV Replication and Virus Production Inhibited in HB611 Cells by Salubrinal Treatment

**Figure 5:**
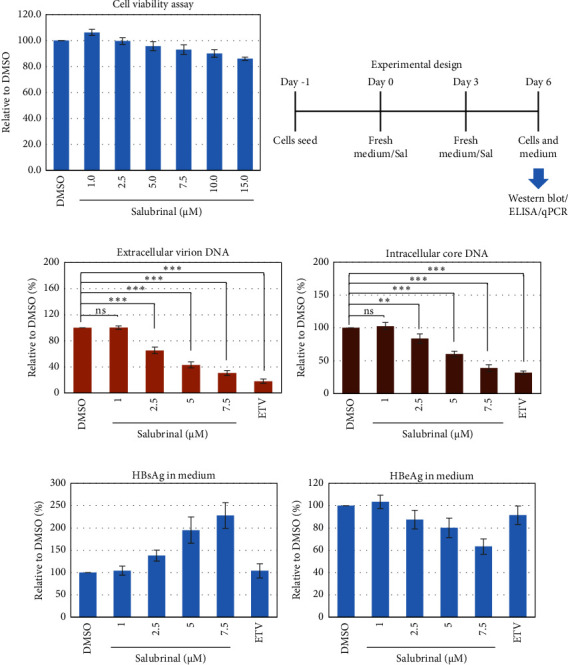
Effect of salubrinal (sal) on the HBV replication and virion production in HB611 cells. HB611 cells were seeded and treated with sal at the indicated concentration. Cells and medium were collected after 6 days of sal treatment for further analysis. (a) Cell viability assay. (b) Experimental design. (c) The extracellular particle-associated DNA quantification. (d) Intracellular core associated DNA quantification. (e) HBsAg and (f) HBeAg ELISA from the medium of the cultured cells. Each of the experiments was conducted at least three independent times and results were presented as the mean ± SD. ^∗∗^ represents statistically significant (*p* < 0.01) and ^∗∗∗^ indicates (*p* < 0.001).

To further validate the results, stably HBV-producing HB611 cells were treated with various concentrations of salubrinal, followed by the quantification of intracellular viral replication and viron production at day 6 using qPCR (Figure 5(b)). Accordingly, the intracellular core-associated DNA and extracellular virion production were significantly decreased by the sal treatment at dose-dependent manner (Figures 5(c) and 5(d)). Interestingly, sal treatment did show no effect on extracellular viron production on day 3 of posttreatment though intracellular DNA level was decreased (data not shown). These results further proved the inhibitory effect of sal on HBV replication and virion production. However, the secretory HBeAg and HBsAg were decreased and increased, respectively, as also found in transfected HepG2 cells (Figures 5(e) and 5(f)).

### 3.5. Effect of Sal and tm on the HBV Core and Large HBsAg Expression

The secretory HBeAg and HBsAg expressions level showed similar patterns by the treatment of tm and sal, respectively, though the reduction level of HBeAg from salubrinal treated cells are prominent (Figures 2(g), 2(h), 4(e), and 4(f)). Therefore, to find out the exact mechanism of HBV down/upregulation by sal and tm, the ER-stress was investigated. Surprisingly, sal stabilized the ER-stress in this system as the grp78 expression level remained stable showed by Western blot analysis from the sal-treated transfected HepG2 cells (Figure 3(a)). Interestingly, Western blot from sal-treated cell lysates showed that the core protein and large HBsAg were significantly reduced (Figure 3(a)). In addition, the cellular grp78, HBV core, and large HBsAg expression levels in HepG2 cells increased by tm treatment as demonstrated by Western blot analysis (Figure 3(b)). These results suggest that sal and tm might affect the core protein and large HBsAg synthesis by modulating ER-stress and thereby reducing the viral replication and virion production.

## 4. Discussion

HBV replication is regulated and controlled by many host cellular factors and innate immune responses [10, 62]. Similarly, pathogenesis, liver injury, and HCC development also depend on viral replication efficiency [63, 64]. Though vaccination has been practiced to prevent HBV infection, sometimes it may fail to protect the individual [65]. Once the infection has occurred, the anti-HBV drugs could minimize the disease severity by reducing the viral replication but the viral genome could not be completely eradicated from the infected patient [66]. On the other hand, drug-resistant HBV has been emerged and circulating throughout the world [1, 67, 68]. Therefore, drugs targeting host cellular factors might be alternative ways to control HBV-induced pathogenesis [28, 32, 62]. In this study, we reported that tunicamycin induced ER-stress in HepG2 cells and increased HBV viral replication. Interestingly, sal inhibited the HBV replication and virion production by reducing large HBsAg and core protein expression in both transfecting HepG2 cells and stably HBV-producing HB611 cells.

ER is one of the most important intracellular organelles associated with the folding, translocation, and posttranslation modification of proteins. ER-stress might be occurred due to the imbalance of ER-surrounding environments by many stimuli including several viruses which finally modulate cell signaling pathways, leading to cell survival or death depending on the severity of the stress level [69, 70]. Several ER-stress transducers such as PERK (protein kinase R-like ER kinase), ATF6 (activating transcription factor 6), and IRE1 (inositol-requiring enzyme 1*α*) are dissociated by the binding of unfolded viral proteins to the cellular grp78. Then, the activated PERK phosphorylates eIF2*α*, thereby leading to the translational attenuation and induction of cellular GADD34 and CHOP [69]. On the other hand, ATF6 dissociated from grp78 and translocated into the Golgi bodies, cleaved and stimulating the expression of chaperones. Besides, released IRE1 from the grp78 dimerized and activated to induce the splicing of XBP1 mRNA, thereby the expression of UPR targets genes occurs [69, 71]. Viral infections may interfere with this ER-stress signaling pathway and regulate the pathogenesis and viral replication. However, the liver is susceptible to ER-stress and the critical roles of ER-stress and UPR-signaling pathways in the development of liver diseases, including viral hepatitis and HCC, have been demonstrated through studies on human liver tissue and animal disease models [72].

The HBV-induced UPR and ER-stress is proven by many *in vivo* and *in vitro* studies [32, 35, 40, 42]. HBx activates the IRE1*α*/XBP1 and ATF6 pathways of the UPR to accelerate HBV replication, thereby contributing to pathogenesis, while the PERK/ATF4 pathway, repressed by HBx, inhibits cell apoptosis, promoting HBV-associated carcinogenesis [72]. Wild-type and mutated large HBsAg have also been reported to be localized in the ER and induced ER-stress, which is associated with the activation of several oncogenic pathways to contribute the HCC development [42, 73, 74]. In addition, Lee et al. demonstrated that mutated HBV core protein-induced ER-stress, which might have an impact on liver disease progression [44]. However, in accordance with these previous reports, our results also demonstrated that HBV replication in transfected HepG2 cells induces ER-stress. A well-known ER-stress inducer, tm, further aggravates the ER stress induced by HBV and thereby upregulated core and large HBsAg expression and viral replication. These results suggested that ER-stress is beneficial for HBV and disease progression.

Core and envelope proteins are the HBV structural proteins and thereby necessary for complete virion formation and release [75–77]. The core protein self-assembles to form a viral capsid and encapsidates the viral pgRNA, and the core particles are enveloped by the surface proteins in ER-Golgi/MVB to be released into the extracellular space [3, 75, 76]. In the current study, the core and large HBsAg are downregulated by sal, an inhibitor of eukaryotic translational initiation factor eIF2*α* [78]. Sal is a well-known drug used for maintaining and/or reducing ER-stress [49]. Accordingly, in our study, sal was able to maintain a stable expression of ER-stress marker gpr78 in HBV-replicating hepatocytes. Therefore, we hypothesized that the sal-mediated reduction of HBV replication might be due to the simultaneous reduction of translation of core and large HBsAg and maintenance of ER homeostasis. Our results also correlated the effect of sal on other viruses as shown by previous studies [79–81]. Sal treatment significantly decreased herpes simplex virus-1 (HSV-1) and Zika virus production in Vero cells virus [79, 80]. Umareddy et al. found that dengue virus growth has been decreased in adenocarcinomic human alveolar basal epithelial cells by the sal treatment in a dose-dependent manner [81]. Based on our results as well as previous reports, it might be hypothesized that ER-stress might be beneficial for several viral replication and pathogenesis including HBV. Further infections and *in vivo* studies need to be performed for detailed mechanisms and clinical outcomes of HBV replication and pathogenesis by modulation of ER dynamics. However, both secretory HBeAg and small HBsAg showed an almost similar pattern of reduced expression from tm- and sal-treated cells by ELISA that might be independent of the effects of these drugs and/or ER-stress. However, it should be mentioned that the initial concentration of tm used to induce ER-stress was somewhat high. However, as the experiment progressed, a lower concentration was used to evaluate indicators of ER-stress (such as grp78 expression), as well as viral replication and virion production. In addition, a limitation of the current study is that no experiments were conducted in primary human hepatocytes and in transgenic mice through de novo infection.

In conclusion, ER-stress aggravated by tm upregulates the core and large HBsAg expression and finally HBV replication and virion production. The sal-mediated inhibition of the HBV replication and virion production might be due to the simultaneous reduction of core and large HBsAg expression and maintaining the ER homeostasis. The results of HBV replication regulation by modulation of ER-stress dynamics would be useful for designing/identifying anti-HBV drugs targeting cellular signaling pathways.

## Data Availability

The data used to support the findings of this study are available from the corresponding author upon reasonable request.
